# Efficacy of denosumab versus alendronate for aromatase inhibitor-associated osteoporosis in postmenopausal breast cancer patients: a retrospective analysis

**DOI:** 10.1186/s12891-025-09280-w

**Published:** 2025-11-12

**Authors:** Yi Zhang, Wenhe Cui, Lei Hou

**Affiliations:** 1 The Eighth Clinical Medical College of Guangzhou, University of Chinese Medicine, Foshan, 528000 Guangdong China; 2 Foshan Hospital of Traditional Chinese Medicine, Foshan, 528000 Guangdong China

**Keywords:** Denosumab, Alendronate, Aromatase inhibitors, Osteoporosis

## Abstract

**Objective:**

Bisphosphonates and denosumab can increase bone mineral density (BMD) and are used to treat osteoporosis caused by aromatase inhibitors (AIs). However, few studies have been conducted on the effects of both on vertebral compression fractures (VCFs). This article aims to compare the effects of alendronate sodium and denosumab injection on the frequency of VCFs in postmenopausal women whose osteoporosis was brought on by AIs treatment for breast cancer.

**Methods:**

A retrospective cohort study was conducted from January 2020 to December 2024, enrolling 121eligible breast cancer patients with aromatase inhibitor-associated osteoporosis from the orthopedic outpatient department of Foshan Hospital of Traditional Chinese Medicine. Patients were divided into two treatment groups: the alendronate group received oral alendronate sodium tablets (70 mg once weekly), while the denosumab group received subcutaneous denosumab injections (60 mg every 6 months). Both groups were supplemented with calcitriol and calcium carbonate/vitamin D3 tablets as baseline therapy. The observation period was 12 months. The following parameters were compared between the two groups before and after treatment: BMD, 25-hydroxy Vitamin D3 (25-OH D3), β-C-terminal telopeptide of type I collagen (β-CTX) and Procollagen I N-Terminal Propeptide (PINP), Visual Analog Scale (VAS) scores and Incidence of VCFs. Statistical analysis was performed using SPSS 27.0.

**Results:**

The study included a total of 121 patients. Post-treatment analysis revealed a significantly higher overall response rate in the denosumab group(*n* = 57) (91.22%) compared to the alendronate group(*n* = 64) (82.81%; *P* < 0.05). Notably, the denosumab group demonstrated superior outcomes in the following two areas: (1) significantly greater improvement in BMD, (2) lower incidence of vertebral compression fractures (both *P* < 0.05). Both treatment groups showed statistically significant improvements in bone metabolism markers following treatment (*P* < 0.01).

**Conclusion:**

Both therapeutic regimens effectively improved BMD in the study population. However, comparative analysis revealed that denosumab injection (60 mg every 6 months) demonstrated significant advantages over weekly alendronate sodium (70 mg) in multiple clinical outcomes. Specifically, the denosumab group showed: (1) greater BMD improvement at all measured skeletal sites, and (2) a significantly lower incidence of VCFs (all *P* < 0.05) in postmenopausal women with aromatase inhibitor-associated osteoporosis.

## Background

 Breast cancer represents the most frequently diagnosed malignancy in women globally, with high morbidity and mortality rates in people over 50 years of age [1]. Approximately 75% of cases are hormone receptor-positive (HR+), characterized by expression of estrogen receptor (ER) and/or progesterone receptor (PR) [2]. Importantly, elevated hormone receptor expression has been significantly associated with an increased risk of distant metastasis [3]. Endocrine therapy constitutes the cornerstone treatment for hormone receptor-positive (HR+) breast cancer. Current clinical guidelines recommend selective estrogen receptor modulators (SERMs; e.g., tamoxifen) as first-line therapy for premenopausal patients, while third-generation AIs (e.g., exemestane, letrozole, anastrozole) represent the standard of care for postmenopausal women [4]. Although these regimens significantly reduce recurrence rates and cancer-specific mortality in HR + early breast cancer (EBC), prolonged administration is associated with clinically significant adverse effects [5, 6]. The skeletal effects of endocrine therapy are mediated through estrogen’s pivotal role in bone homeostasis. In premenopausal women, physiological estrogen levels maintain bone integrity through balanced regulation of osteoblast-mediated bone formation and osteoclast-mediated resorption. The postmenopausal state, characterized by ovarian failure and consequent estrogen deficiency, disrupts this equilibrium through multiple mechanisms: (1) impaired osteocyte function, (2) upregulated osteoclast activity, and (3) diminished bone mineralization capacity. These pathological changes collectively lead to accelerated bone loss, microarchitectural deterioration, and compromised biomechanical strength, culminating in osteoporosis and increased susceptibility to fragility fractures [7]. Other clinically significant risk factors, including metabolic comorbidities such as type 2 diabetes mellitus and obesity (BMI ≥ 30 kg/m²), must be carefully evaluated in conjunction with primary treatment considerations [8, 9].

Current clinical guidelines recommend both oral and intravenous bisphosphonates as effective therapeutic interventions for managing aromatase inhibitor-associated bone loss, with demonstrated clinical efficacy in preserving BMD. The pharmacological action of bisphosphonates is mediated through their high affinity for hydroxyapatite crystals in the bone mineral matrix. Following incorporation into the bone structure, these compounds are selectively taken up by osteoclasts during bone resorption, where they induce cellular apoptosis through inhibition of the mevalonate pathway, thereby effectively suppressing excessive bone turnover [10]. Bisphosphonates have emerged as first-line pharmacologic therapy for osteoporosis owing to their potent antiresorptive properties and demonstrated efficacy in increasing BMD [11].

Denosumab is a fully human monoclonal antibody (IgG2 subtype) that functions as a potent anti-resorptive agent through its high-affinity binding to receptor activator of nuclear factor kappa-B ligand (RANKL). The therapeutic mechanism involves: (1) neutralization of circulating RANKL, (2) blockade of RANKL-RANK interaction, and (3) subsequent modulation of the OPG-RANKL-RANK signaling pathway. This targeted action results in three principal clinical effects: (i) suppression of osteoclast differentiation and activation, (ii) enhancement of BMD through reduced bone resorption, and (iii) alleviation of bone pain. Clinically, denosumab is approved for two major indications: treatment of postmenopausal osteoporosis and prevention of skeletal-related events in patients with metastatic breast cancer to bone [12].

From a pharmacokinetic perspective, denosumab exhibits unique metabolic characteristics. As a monoclonal antibody, it undergoes catabolism via proteolytic degradation without hepatic metabolism. Importantly, renal impairment does not significantly alter its pharmacokinetic profile, as demonstrated in clinical pharmacokinetic studies. This distinctive property makes denosumab particularly suitable for patients with compromised renal function [13]. Clinical studies have consistently demonstrated the superior efficacy of denosumab in augmenting BMD compared to other antiresorptive agents, with significant improvements observed at key skeletal sites including the lumbar spine (LS), total hip (TH), and femoral neck (FN) [14]. Based on its demonstrated clinical efficacy, denosumab received regulatory approval for osteoporosis treatment in multiple jurisdictions [15].

Despite the widespread clinical use of both denosumab and bisphosphonates (e.g., alendronate) for osteoporosis management, there remains a paucity of rigorous comparative studies evaluating their relative efficacy in two key domains: (1) BMD improvement, and (2) fracture prevention. To address this critical knowledge gap, we designed this comparative effectiveness study to systematically evaluate these two antiresorptive therapies in breast cancer patients with aromatase inhibitor-induced osteoporosis. Our primary objectives are to compare their effects on: (i) BMD changes at lumbar spine, femoral neck, and total body, (ii) serum bone turnover markers (including β-CTX and P1NP) and 25-OH D3, (iii) incidence of VCFs over a 12-month treatment period.

## Patients and methods

### Study subjects

**Fig. 1:**
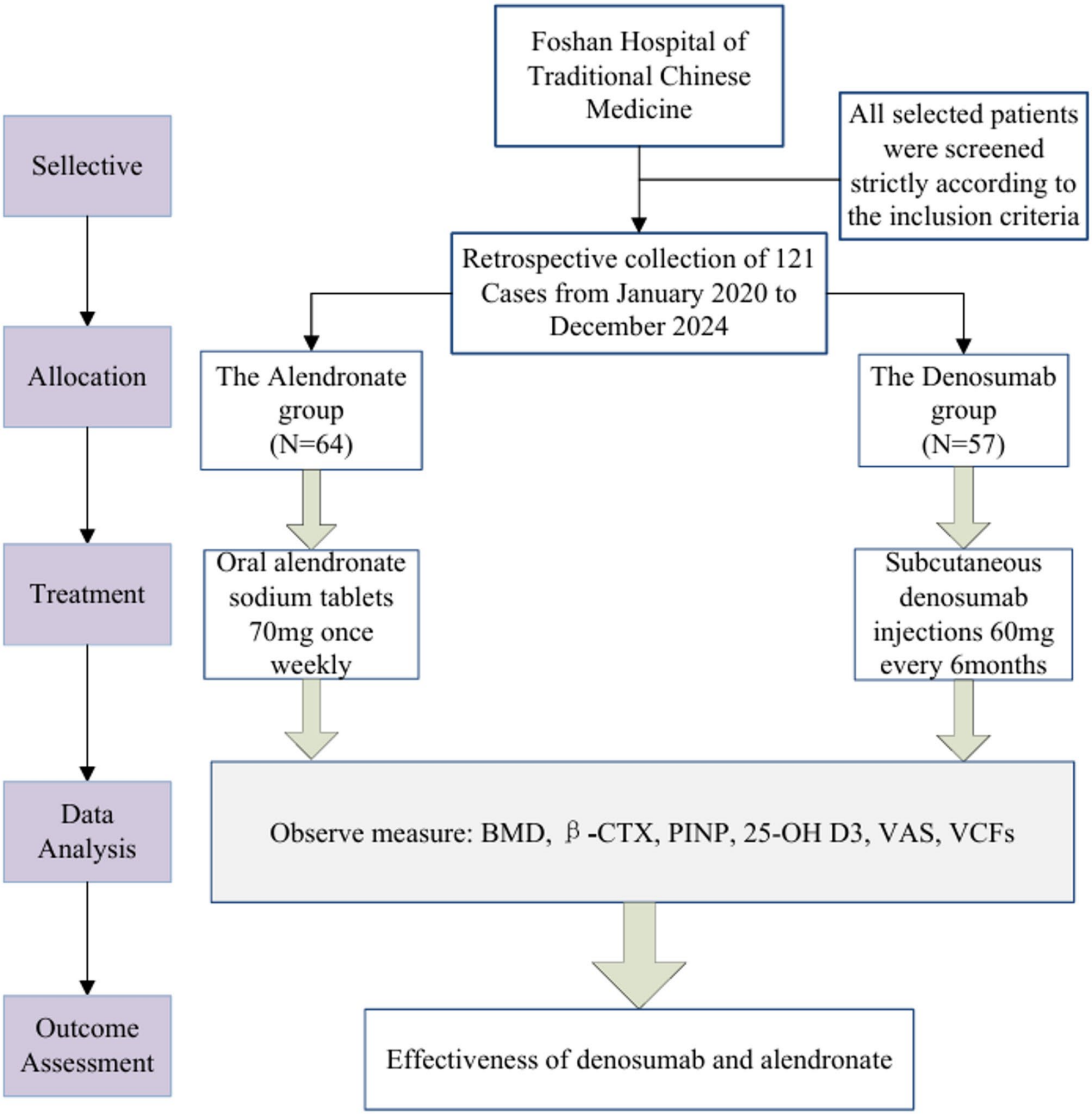
Flow of study design

We conducted a retrospective cohort analysis of 121 breast cancer patients who developed aromatase inhibitor-induced osteoporosis and were treated at the Orthopedics Outpatient Clinic of Foshan Hospital of Traditional Chinese Medicine from January 2020 to December 2024. All participants received baseline therapy with oral calcitriol (0.25 µg/day) and calcium carbonate/vitamin D3 (600 mg/400 IU daily). Based on their osteoporosis treatment regimen, patients were stratified into two groups: The alendronate group (*n* = 64) received weekly oral alendronate (70 mg), and the denosumab group (*n* = 57) received subcutaneous denosumab (60 mg every 6 months). Diagnostic Criteria 1. Histologically confirmed diagnosis of invasive epithelial breast carcinoma. Postmenopausal status, as rigorously defined by the *NCCN Clinical Practice Guidelines in Oncology: Breast Cancer* (2018 version) [16]0.2. Hormone receptor (HR) status was determined according to the *American Society of Clinical Oncology/College of American Pathologists (ASCO/CAP) Guideline Recommendations for Immunohistochemical Testing of Estrogen and Progesterone Receptors in Breast Cancer* (2010 version). HR positivity was defined as ≥ 1% nuclear staining for either estrogen receptor (ER) or progesterone receptor (PR) in invasive tumor cells, as assessed by validated immunohistochemical methods [17]. 3. BMD was evaluated according to World Health Organization (WHO) diagnostic criteria for osteoporosis in postmenopausal women. Measurements were obtained at two anatomical sites: (1) lumbar spine (L1-L4) via anteroposterior dual-energy X-ray absorptiometry (DXA), (2) total hip (including femoral neck), and (3) total body. Results were interpreted using T-scores, with the following WHO classification thresholds: normal (T-score ≥ −1.0), osteopenia (−2.5 < T-score < −1.0), and osteoporosis (T-score ≤ −2.5) [18]. The study design flow is illustrated in Fig. 1. Inclusion Criteria: Eligible patients met al.l of the following criteria: (1) Histologically confirmed invasive breast cancer with hormone receptor positivity. (2) Pathological stage I-IIIA disease according to the AJCC 8th edition staging system. (3) Age ≤ 75 years with preserved hepatic and renal function. (4) Completion of primary treatment, including definitive breast surgery, adjuvant chemotherapy (if indicated), and radiotherapy (if indicated). (5) Current treatment with adjuvant endocrine therapy. (6) Postmenopausal status, defined as either natural menopause or induced menopause. (7) Minimum antiresorptive therapy exposure ≥ 12 months of weekly oral alendronate (70 mg), or ≥ 2 doses of subcutaneous denosumab (60 mg every 6 months). (8) Complete availability of baseline and follow-up clinicopathological data, Serial BMD measurements, and signed informed consent documents. Exclusion Criteria: Patients were excluded if they met any of the following conditions: (1) Presence of metastatic breast cancer. (2) Chronic glucocorticoid use (>3 months cumulative use within the past year). (3) Significant hepatic dysfunction or renal impairment. (4) Concurrent metabolic bone diseases (including but not limited to: Primary hyperparathyroidism, Paget’s disease of bone, and Osteogenesis imperfecta. (5) Severe comorbidities include psychiatric conditions that may compromise pain assessment reliability.

### Methods

Both treatment groups maintained their prescribed endocrine therapy regimens throughout the study period, with all participants receiving standardized baseline supplementation (calcitriol 0.25 µg/day plus calcium carbonate 600 mg/day). The alendronate group (*n* = 64) was administered oral alendronate sodium (Fosamax^®^; Hangzhou MSD Pharmaceutical Co., Ltd.; NMPA approval no. J20140144) 70 mg once weekly. The denosumab group (*n* = 57) received subcutaneous denosumab (Prolia^®^; 60 mg) every 6 months. The comparative treatment period was 12 months, and we evaluated pre- and post-treatment indicators: BMD, β-CTX, P1NP, 25-(OH) D3, radiographically confirmed VCF incidence, and VAS pain scores.

### Efficacy evaluation criteria

Therapeutic Efficacy Assessment Criteria for Osteoporosis: Markedly Effective Response: Complete resolution or near-complete resolution (≥ 80% reduction) of baseline pain symptoms, significant improvement in BMD by dual-energy X-ray absorptiometry (DXA). Effective response: Clinically meaningful pain reduction, Maintenance of baseline BMD (± 1% change) or modest improvement, No sleep disturbance attributable to persistent pain. Ineffective response: failure to achieve ≥ 50% pain reduction, no significant BMD improvement, and no measurable improvement in quality of life parameters [10].

### Statistical methods

All statistical analyses were performed using SPSS version 27.0, with a two-tailed significance threshold set at α = 0.05. Normally distributed count data were expressed as frequencies and analyzed using the chi-square test. Measurement data were assessed for normality (Shapiro-Wilk test) and homogeneity of variance (F-test). Normally distributed measurement data were presented as mean ± standard deviation (x̄ ± s). Intergroup comparisons of normally distributed measurement data were performed using the independent t-test (Student’s t-test for equal variances; Welch’s t-test for unequal variances). Intragroup comparisons before and after treatment were conducted using the paired t-test. Non-normally distributed continuous variables were expressed as medians and interquartile ranges [M (P25, P75)]. Intergroup comparisons were performed using the Mann–Whitney U test, and intragroup comparisons were conducted using the Wilcoxon signed-rank test.

*P* < 0.05 was considered statistically significant.

## Results

### Baseline characteristics

Baseline demographic and clinical characteristics, including age, body mass index (BMI), menopausal duration, and duration of AIs therapy, were well-balanced between the two treatment groups (all *P* > 0.05), demonstrating comparable baseline profiles (Table 1).

**Table 1:** Comparison of general data between the two groups (x ± s)

Grope	Year	BMI/(g/cm2)	Menopause period (year)		Taking Ais time (month)
Alendronate(*n* = 64)	59.58 ± 6.01	22.49± 2.71	9.02 ± 4.37	22.81 ± 10.04	
Denosumab(*n* = 57)	61.16 ± 5.07	22.62 ± 3.44	8.88 ± 3.83	24.07 ± 9.86	
*t*	1.552	0.232	0.184	0.694	
*P*	0.123	0.817	0.853	0.489	

### Comparison of BMD before and after treatment

#### Lumbar spine BMD T-Score comparison

Initial assessment revealed no statistically significant intergroup difference in lumbar spine BMD T-score (*P* = 0.076), confirming appropriate baseline matching between cohorts. Following the intervention period, an independent samples t-test demonstrated significantly greater improvement in lumbar spine BMD T-score in the denosumab group compared to the alendronate group (*P* = 0.003). Paired t-tests indicated significant within-group improvements from baseline in both treatment arms(*P*<0.001) (Table 2).

**Table 2:** Changes in bone mineral density T-values, bone metabolic indexes and 25(OH)D3 before and after treatment in the two groups

Group	lumbar Spine BMD T-score (x ± s, g/cm2)		femoral neck BMD T-score (x ± s, g/cm2)		total body BMD T-score (x ± s, g/cm2)	β-CTX [M (P25, P75) ng/mL]	PINP [M (P25, P75) ng/mL]	25-OH D3[M (P25, P75) ng/mL]
Alendronate(*n* = 64)								
before treatment		−2.96 ± 0.77		−2.68 ± 0.68	−2.77 ± 0.84	60.92(0.32, 0.78)	62.50(36.44, ,56.08)	59.22(16.09, 26.347)
after treatment		−2.72 ± 0.78		−2.55 ± 0.72	−2.63 ± 0.80	64.31(0.30, 0.68)	66.13(47.43, 66.27)	59.27(19.77, 38.83)
*t/Z/U*		7.232		4.888	5.785	−3.301	−5.026	−6.52
*P*-value		<0.001		<0.001	<0.001	<0.001	<0.001	<0.001
Denosumab(*n* = 57)								
before treatment		−3.47 ± 0.99		−3.15 ± 0.95	−3.20± 1.08	61.09(0.38, 0.76)	59.24(33.77, 56.79)	63.00(16.67, 27.41)
after treatment		−3.19± 0.94		−3.01± 0.92	−3.05 ± 1.04	57.28(0.27, 0.64)	55.25(42.45, 62.41)	62.95(20.83, 33.98)
*t/Z/U*		7.575		4.058	7.981	−4.308	−4.517	−5.097
*P*-value		<0.001		<0.001	<0.001	<0.001	<0.001	<0.001
*t1/Z1/U1*		2.902		1.109	1.456	1819	1723	1710
*P1*-value		0.076		0.077	0.116	0.979	0.554	0.554
*t2/Z2/U2*		3.027		3.088	2.468	1612	1496	1713
*P2*-value		0.003		0.03	0.026	0.271	0.089	0.564

*t1/Z1/U1, P1*-value means Comparison of the two groups before treatment; *t2/Z2/U2, P2*-value means Comparison of the two groups after treatment

#### Femoral neck BMD T-Score comparison

Initial measurements showed comparable femoral neck BMD T-score between groups (*P* = 0.077), confirming balanced baseline characteristics. After 12 months of treatment, the denosumab group demonstrated significantly greater improvement in femoral neck BMD T-score compared to the alendronate group (*P* = 0.005). The BMD T-score of the femoral neck changed significantly in both groups after treatment(*P*<0.001) (Table 2).

#### Total body BMD T-Score comparison

Initial total body BMD T-score measurements showed no significant intergroup differences (*P* = 0.116), confirming comparable baseline characteristics between treatment arms. Following the intervention period, independent samples t-test analysis revealed statistically superior total body BMD T-score improvements in the denosumab group versus the alendronate group (*P* = 0.026). Paired t-tests demonstrated significant improvements from baseline in both groups (*P*<0.001) (Table 2).

#### Comparison of 25-OH D3 levels before and after treatment

No significant difference was observed in baseline serum 25-OH D3 levels between the denosumab groups (*P* = 0.554), confirming comparable baseline vitamin D status. The Mann-Whitney U test showed no statistically significant difference in post-treatment 25-OH D3 levels between groups (*P* = 0.564). Wilcoxon signed-rank tests revealed significant increases from baseline in both treatment arms(*P*<0.001) (Table 2).

#### Comparison of PINP levels before and after treatment

Initial serum PINP levels were comparable between groups (*P* = 0.554), indicating balanced baseline bone formation status. The Mann-Whitney U test revealed no significant difference in post-treatment PINP levels between groups (*P* = 0.089). Wilcoxon signed-rank tests demonstrated significant reductions from baseline in both treatment arms (*P*<0.001) (Table 2).

#### Comparison of β-CTX levels before and after treatment

No significant difference was observed in baseline serum β-CTX levels between the treatment groups (*P* = 0.979), indicating comparable baseline bone resorption activity. The Mann-Whitney U test demonstrated no statistically significant difference in post-treatment β-CTX levels between groups (*P* = 0.271). Wilcoxon signed-rank tests revealed significant reductions in β-CTX levels from baseline in both groups (*P*<0.001) (Table 2).

#### Comparison of VAS scores before and after treatment

Initial VAS pain scores demonstrated no significant difference between groups (*P* = 0.665), confirming comparable baseline pain levels. Independent samples t-test revealed no statistically significant difference in post-treatment VAS scores between groups (*P* = 0.892). Paired t-tests demonstrated significant pain reduction in both treatment arms (*P*<0.001) (Table 3).

**Table 3:** Comparison of VAS scores between the two groups before and after treatment(x ± s)

Group	Before treatment	After 6 months of treatment	After 12 months of treatment	t	*P*
Alendronate(*n* = 64)	6.22± 0.15	5.36± 0.14	3.56 ± 0.12	19.914	<0.001
Denosumab(*n* = 57)	6.33± 0.21	5.33± 0.16	3.59 ± 0.13	18.682	<0.001
*t*	0.434	0.115	0.136		
*P*	0.665	0.906	0.892		

#### Comparison of vertebral fracture incidence before and after treatment

In the alendronate group, post-treatment thoracic compression fractures occurred in 3 cases and lumbar compression fractures in 6 cases (total fracture rate: 12.5%). The denosumab group reported 1 thoracic and 3 lumbar compression fractures (total fracture rate: 7.017%). Fisher’s exact test demonstrated a significantly lower fracture incidence in the denosumab group (*P* < 0.05) (Table 4).

**Table 4:** The incidence of vertebral fractures before and after treatment in the two groups (cases %)

Group	Case	thoracic fracture	lumbar fracture	total rate
Alendronate	64	3(4.69)	6(9.38)	9(14.06)
Denosumab	57	1(1.75)	3(5.26)	4(7.02)
*X*^*2*^				12.00
*P*				0.002

#### Efficacy evaluation before and after treatment

The denosumab group exhibited a higher treatment efficacy rate (91.22%) compared to the alendronate group (82.81%). Fisher’s exact test confirmed the superiority of the denosumab group (*P* < 0.05) (Table 5).

**Table 5:** Clinical efficacy evaluation of the two groups of patients after treatment (cases/%)

Group	Case		Significant effect	Effective	Ineffective	effective rate(%)
Alendronate		64	36	17	11	53(82.81)
Denosumab		57	37	15	5	52(91.22)
*X*^*2*^						4.571
*P*						0.03

## Discussion

Endocrine therapy, utilizing estrogen receptor antagonists and AIs, has become a cornerstone of postoperative breast cancer management by reducing estrogen levels and modifying the tumor microenvironment to inhibit neoplastic growth [19]. Clinical evidence from multiple randomized controlled trials has established that completing a standard 5-year course of adjuvant endocrine therapy in hormone receptor-positive early breast cancer (HR [+] EBC) patients is associated with significant Survival Benefit and durable Protection [20]. Furthermore, multiple clinical trials have confirmed that extending endocrine therapy to up to 10 years further decreases the recurrence and mortality rates of HR [+] EBC. The third-generation AIs have been established as the first-line endocrine therapy for postmenopausal women with hormone receptor-positive (HR+) breast cancer [21].

Despite their oncologic efficacy, endocrine therapies adversely impact bone health through estrogen suppression, leading to accelerated bone loss and significantly elevated fracture risk in breast cancer patients [5, 22]. AIs therapy in HR[+] breast cancer patients is associated with 17–22% 5-year fracture risk, mediated through hypoestrogenism-induced bone turnover imbalance (40–60% CTX elevation) and preferential trabecular bone loss (5–8% annual lumbar spine BMD decline) [23]. A meta-analysis revealed a positive correlation between prolonged AI treatment duration and increased fracture incidence [24]. Goss et al. (MA.17R trial) reported that high-risk patients on extended AI therapy (10 years) face progressive annual fracture risk increases (2–4%) [25]. HR-pQCT analysis by Kuba et al. demonstrated significant 12-month AI-induced deterioration in postmenopausal breast cancer patients, including trabecular BMD loss, cortical thinning, and reduced bone strength [26]. Moreover, all AIs carry significantly higher risks of osteoporosis and fractures versus tamoxifen, with consistent effects across anastrozole, letrozole, and exemestane [5, 27].

Current guidelines recommend combining lifestyle modifications (calcium/vitamin D, exercise) with anti-resorptives (bisphosphonates/denosumab) for bone protection in AIs treated patients [28]. Bisphosphonates and denosumab are essential for maintaining bone health in this population. A recent study reported that early postmenopausal women with ER[+] breast cancer receiving AI therapy combined with anti-resorptive treatment exhibited significant increases in femoral and lumbar spine BMD (6.28% and 7.79%, respectively) after 24 months [29]. Anti-resorptive therapy markedly improves BMD in postmenopausal women with early breast cancer treated with AIs, thereby mitigating bone loss.

Bisphosphonates play a crucial role in regulating bone metabolism. They are effective in increasing BMD, reducing the risk of fractures, and they generally have favorable safety profiles [30]. By reducing levels of neuropeptides such as substance P (SP) and calcitonin gene-related peptide (CGRP), as well as inflammatory cytokines like tumor necrosis factor-alpha (TNF-α), bisphosphonates effectively alleviate pain in patients with osteoporosis and improve their overall quality of life. However, clinical caution is necessary, as bisphosphonates are contraindicated for patients with renal impairment (specifically, those with a creatinine clearance of less than 35 mL/min). Additionally, renal function should be closely monitored in elderly patients [31].

Recent years have seen a rise in the clinical application of denosumab. A literature review found that, compared to oral bisphosphonates, denosumab enhances patient preference and adherence, while providing a cost-effective therapeutic strategy, especially for elderly patients and those at high risk for osteoporosis [32]. Meta-analyses show that denosumab leads to greater gains in BMD compared to bisphosphonates after 12 months [33]. Denosumab shows promise in preventing BRCA1-mutated breast cancer by inhibiting bone metastasis and providing additional anticancer effects, significantly improving patient prognosis [34].

Denosumab has been recognized as an effective and safe treatment for preventing major osteoporotic fractures, especially vertebral and femoral fractures [35, 36]. In the prospective ABCSG-18 trial, denosumab (administered at a dose of 60 mg biannually) was found to reduce the risk of clinical fractures in postmenopausal breast cancer patients who were receiving AIs, compared to those in the vehicle group. Importantly, this treatment did not result in any additional adverse effects. Furthermore, denosumab doubled the time to the first fracture and significantly increased BMD at the lumbar spine, total hip, and femoral neck [37]. A network meta-analysis showed that after 12 months of treatment, denosumab decreased the risk of vertebral fractures by up to 85% compared to the vehicle. Multiple studies confirm that denosumab is the most effective therapy for preventing vertebral fractures [38–40].

Bone remodeling status can be evaluated using metabolic markers. PINP and β-CTX serve as specific markers for bone formation and resorption, respectively [41]. PINP is a biomarker that shows high sensitivity and specificity. It is less affected by hormonal changes and provides insight into osteoblast-mediated collagen synthesis, making it valuable for diagnosing metabolic bone diseases and renal insufficiency. On the other hand, β-CTX is the most commonly used marker for collagen degradation. It is an isomer of α-CTX and is closely related to the intensity of bone resorption. β-CTX responds quickly and sensitively to anti-resorptive therapies and can help predict the severity of abnormal bone turnover. 25-OH D is the main circulating form of vitamin D and accurately reflects the body’s vitamin D status. The liver hydroxylates vitamin D to produce 25-OH D, which is then activated to 1,25-OH D in the kidneys.

This study compared the effectiveness of alendronate and denosumab in treating aromatase inhibitor-induced osteoporosis in postmenopausal breast cancer patients. After one year of treatment, both groups showed increases in BMD at the lumbar spine, femoral neck, and total body, confirming that both medications are effective. Biochemical analyses indicated elevated levels of 25-OH D3 and P1NP, along with reduced β-CTX levels in both groups. Among the 64 patients receiving alendronate, three experienced thoracic VCFs, and five had lumbar VCFs. In the denosumab group, consisting of 57 patients, there was one thoracic VCF and three lumbar VCFs, suggesting a lower incidence of fractures with denosumab. Both treatments improved bone metabolic parameters by suppressing bone turnover and enhancing BMD.

To summarize, the relationship between breast cancer and bone health requires strategies to reduce skeletal complications during treatment. As researchers work to understand the mechanisms behind both acquired and inherent resistance to endocrine therapies and CDK4/6 inhibitors, it is essential to develop new endocrine agents that offer higher efficacy with fewer side effects [42]. Future research could improve tools for assessing fracture risk and lead to the development of more effective therapies.

This study has several limitations: (1) The timing of anti-osteoporotic drug initiation post-AI therapy may influence outcomes; (2) As a single-center retrospective study, the collection of patient data was non-randomized and potentially incomplete; (3) The small sample size may introduce selection bias, limiting the generalizability of findings; (4) The short follow-up period (one year) is insufficient to evaluate long-term efficacy and safety; (5) Rare adverse events (e.g., osteonecrosis of the jaw) were not fully monitored. Future research should increase sample sizes, extend follow-up durations, and evaluate long-term adverse effects to thoroughly assess the clinical effectiveness of denosumab and alendronate.

## Conclusion

Both alendronate and denosumab are effective in treating AI-induced osteoporosis in breast cancer patients by improving BMD and modulating bone metabolism. Denosumab offers a reduced risk of VCFs.

### Authors’ contributions

YZ contributed to the design of this article, conducted the statistical analyses, and drafted the manuscript. WHC assisted with the statistical analyses. LH provided critical revisions for the manuscript. All authors reviewed and approved the final manuscript.

## Data Availability

If someone wants to request the data from this study, he/she should contact Yi Zhang ([1940233878@qq.com](mailto:1940233878@qq.com)).

## References

[1] Kim J, Harper A, McCormack V, et al. Global patterns and trends in breast cancer incidence and mortality across 185 countries. Nat Med. 2025;31(4):1154–62. 39994475 10.1038/s41591-025-03502-3

[2] Zattarin E, Leporati R, Ligorio F, et al. Hormone receptor loss in breast cancer: molecular Mechanisms, clinical Settings, and therapeutic implications. Cells. 2020;9(12):2644. Published 2020 Dec 9. 33316954 10.3390/cells9122644 PMC7764472

[3] Chen L, Zhang Y, Li J, Kalmar A. Efficacy and safety of traditional Chinese medicine in managing bone loss post-endocrine therapy in hormone receptor-positive breast cancer patients. Medicine (Baltimore). 2024;103(41):e39961. 39465878 10.1097/MD.0000000000039961 PMC11479473

[4] Korde LA, Somerfield MR, Carey LA, et al. Neoadjuvant chemotherapy, endocrine therapy, and targeted therapy for breast cancer: ASCO guideline. J Clin Oncol. 2021;39(13):1485–505. 33507815 10.1200/JCO.20.03399 PMC8274745

[5] Xu J, Cao B, Li C, Li G. The recent progress of endocrine therapy-induced osteoporosis in estrogen-positive breast cancer therapy. Front Oncol. 2023;13:1218206. Published 2023 Jul 7. 37483519 10.3389/fonc.2023.1218206 PMC10361726

[6] Waqas K, Lima Ferreira J, Tsourdi E, Body JJ, Hadji P, Zillikens MC. Updated guidance on the management of cancer treatment-induced bone loss (CTIBL) in pre- and postmenopausal women with early-stage breast cancer. J Bone Oncol. 2021;28:100355. Published 2021 Mar 18. 33948427 10.1016/j.jbo.2021.100355 PMC8080519

[7] Pellegrino A, Tiidus PM, Vandenboom R. Mechanisms of estrogen influence on skeletal muscle: mass, regeneration, and mitochondrial function. Sports Med. 2022;52(12):2853–69. 35907119 10.1007/s40279-022-01733-9

[8] Roomi AB, Mahdi Salih AH, Noori SD, Nori W, Tariq S. Evaluation of bone mineral Density, serum Osteocalcin, and osteopontin levels in postmenopausal women with type 2 diabetes Mellitus, with/without osteoporosis. J Osteoporos. 2022;2022:1437061. Published 2022 Feb 14. 35198139 10.1155/2022/1437061 PMC8860540

[9] Roomi AB, Nori W, Al-Badry SH. The value of serum adiponectin in osteoporotic women: does weight have an effect? J Obes. 2021;2021:5325813. Published 2021 Nov 9. 34796028 10.1155/2021/5325813 PMC8595024

[10] Nakatsukasa K, Koyama H, Ouchi Y, et al. Effects of denosumab on bone mineral density in Japanese women with osteoporosis treated with aromatase inhibitors for breast cancer. J Bone Miner Metab. 2019;37(2):301–6. 29520506 10.1007/s00774-018-0917-0

[11] Kennel KA, Drake MT. Adverse effects of bisphosphonates: implications for osteoporosis management. Mayo Clin Proc. 2009;84(7):632–8. 19567717 10.1016/S0025-6196(11)60752-0 PMC2704135

[12] Giannakeas V, Cadarette SM, Ban JK, Lipscombe L, Narod SA, Kotsopoulos J. Denosumab and breast cancer risk in postmenopausal women: a population-based cohort study. Br J Cancer. 2018;119(11):1421–7. 30420611 10.1038/s41416-018-0225-4 PMC6265331

[13] Wang W. Effects and progress of anti-osteoporosis drugs [J]. Chin J Med. 2021;56(11):1189–92.

[14] Jian L, Dongwen L, Da L, et al. Research progress on Zoledronic acid versus denosumab in the treatment of primary osteoporosis [J]. Med Res Trauma Treat. 2023;36(03):317–23.

[15] Burden AM, Tadrous M, Calzavara A, Cadarette SM. Uptake and characteristics of Zoledronic acid and denosumab patients and physicians in Ontario, Canada: impact of drug formulary access. Osteoporos Int. 2015;26:1525–33. 25603794 10.1007/s00198-014-3023-8

[16] Dandan M, Dan Z. Update of the American society of clinical oncology clinical practice guidelines for adjuvant chemotherapy and targeted therapy in early breast cancer (2018 Edition) [J]. Chin J Breast Disease (Electronic Edition). 2018;12(04):256.

[17] Li S, Xiaowei Q. Guidelines from the American society of clinical oncology and the college of American pathologists for immunohistochemical detection of Estrogen/Progesterone receptors in breast cancer [J]. Chin J Breast Disease (Electronic Edition). 2011;5(03):385–7.

[18] Zhang Zhenlin YH. Key points analysis of the guidelines for the diagnosis and treatment of primary osteoporosis (2022 Edition) [J]. J Intern Crit Care. 2024;30(4):289–93.

[19] Yao S, Laurent CA, Roh JM, et al. Serum bone markers and risk of osteoporosis and fragility fractures in women who received endocrine therapy for breast cancer: a prospective study. Breast Cancer Res Treat. 2020;180(1):187–95. 31912328 10.1007/s10549-019-05518-z PMC7171589

[20] Early Breast Cancer Trialists’ Collaborative Group (EBCTCG). Aromatase inhibitors versus tamoxifen in early breast cancer: patient-level meta-analysis of the randomised trials. Lancet. 2015;386(10001):1341–52. 26211827 10.1016/S0140-6736(15)61074-1

[21] Davies C, Pan H, Godwin J et al. Long-term effects of continuing adjuvant Tamoxifen to 10 years versus stopping at 5 years after diagnosis of oestrogen receptor-positive breast cancer: ATLAS, a randomised trial [published correction appears in lancet. 2013;381(9869):804. 10.1016/S0140-6736(12)61963-1 PMC3596060 23219286

[22] Goel B, Virmani T, Jain V, Kumar G, Sharma A, Al Noman A. Unveiling the link between breast cancer treatment and osteoporosis: implications for anticancer therapy and bone health. Biomed Res Int. 2024;2024:5594542. Published 2024 Nov 14. 39574432 10.1155/2024/5594542 PMC11581800

[23] Deveza LA, Kraus VB, Collins JE, et al. Association between biochemical markers of bone turnover and bone changes on imaging: data from the osteoarthritis initiative. Arthritis Care Res (Hoboken). 2017;69(8):1179–91. 27723280 10.1002/acr.23121 PMC5385286

[24] Goldvaser H, Barnes TA, Šeruga B, et al. Toxicity of extended adjuvant therapy with aromatase inhibitors in early breast cancer: a systematic review and meta-analysis. J Natl Cancer Inst. 2018. 10.1093/jnci/djx141. 28922781 10.1093/jnci/djx141

[25] Goss PE, Ingle JN, Pritchard KI, et al. Extending aromatase-inhibitor adjuvant therapy to 10 years. N Engl J Med. 2016;375(3):209–19. 27264120 10.1056/NEJMoa1604700 PMC5024713

[26] Kuba S, Watanabe K, Chiba K, et al. Adjuvant endocrine therapy effects on bone mineral density and microstructure in women with breast cancer. J Bone Miner Metab. 2021;39(6):1031–40. 34191126 10.1007/s00774-021-01239-w

[27] Chen S, Bo L, Lv D, Ma F. Bone safety profile of steroidal aromatase inhibitor in comparison to nonsteroidal aromatase inhibitors in postmenopausal women with breast cancer: a network meta-analysis. Breast Care. 2022;17(4):391–402. 36156912 10.1159/000523695 PMC9453661

[28] Diana A, Carlino F, Giunta EF, et al. Cancer Treatment-Induced bone loss (CTIBL): state of the Art and proper management in breast cancer patients on endocrine therapy. Curr Treat Options Oncol. 2021;22(5):45. Published 2021 Apr 16. 33864145 10.1007/s11864-021-00835-2 PMC8052225

[29] Mugnier B, Goncalves A, Daumas A, et al. Prevention of aromatase inhibitor-induced bone loss with anti-resorptive therapy in post-menopausal women with early-stage breast cancer. Osteoporos Int. 2023;34(4):703–11. 36715715 10.1007/s00198-023-06683-0

[30] Ayers C, Kansagara D, Lazur B, Fu R, Kwon A, Harrod C. Effectiveness and safety of treatments to prevent fractures in people with low bone mass or primary osteoporosis: a living systematic review and network meta-analysis for the American college of physicians [published correction appears in ann intern Med. 2023;176(6):884. doi: 10.7326/L23-0105]. Ann Intern Med. 2023;176(2):182–95. 36592455 10.7326/M22-0684

[31] Chinese Gerontological Health Medicine Research Association, Geriatric Pain Diseases Branch. Chinese expert consensus on Diagnosis, treatment and management of geriatric osteoporotic pain (2024 Edition) [J]. Chin J Pain Med. 2024;30(04):241–50.

[32] Morizio P, Burkhart JI, Ozawa S, Denosumab. A unique perspective on adherence and Cost-effectiveness compared with oral bisphosphonates in osteoporosis patients. Ann Pharmacother. 2018;52(10):1031–41. 29616561 10.1177/1060028018768808

[33] obayashi T, Morimoto T, Ito K, Mawatari M, Shimazaki T. Denosumab vs. bisphosphonates in primary osteoporosis: a meta-analysis of comparative safety in randomized controlled trials. Osteoporos Int. 2024;35(8):1377–93. 38733394 10.1007/s00198-024-07118-0

[34] Ishikawa T. Differences between zoledronic acid and denosumab for breast cancer treatment. J Bone Miner Metab. 2023;41(3):301–6. 36879056 10.1007/s00774-023-01408-z

[35] Galvano A, Scaturro D, Badalamenti G, et al. Denosumab for bone health in prostate and breast cancer patients receiving endocrine therapy? A systematic review and a meta-analysis of randomized trials. J Bone Oncol. 2019;18:100252. Published 2019 Jul 16. 31440444 10.1016/j.jbo.2019.100252 PMC6700425

[36] Händel MN, Cardoso I, von Bülow C, et al. Fracture risk reduction and safety by osteoporosis treatment compared with placebo or active comparator in postmenopausal women: systematic review, network meta-analysis, and meta-regression analysis of randomised clinical trials. BMJ. 2023;381:e068033. Published 2023 May 2. 37130601 10.1136/bmj-2021-068033 PMC10152340

[37] Gnant M, Pfeiler G, Dubsky PC, et al. Adjuvant denosumab in breast cancer (ABCSG-18): a multicentre, randomised, double-blind, placebo-controlled trial. Lancet. 2015;386(9992):433–43. 26040499 10.1016/S0140-6736(15)60995-3

[38] Nicolopoulos K, Moshi MR, Stringer D, Ma N, Jenal M, Vreugdenburg T. The clinical effectiveness of denosumab (Prolia^®^) in patients with hormone-sensitive cancer receiving endocrine therapy, compared to bisphosphonates, selective Estrogen receptor modulators (SERM), and placebo: a systematic review and network meta-analysis. Arch Osteoporos. 2023;18(1):18. Published 2023 Jan 10. 36624318 10.1007/s11657-023-01211-3

[39] Diab DL, Watts NB. The use of denosumab in osteoporosis - an update on efficacy and drug safety. Expert Opin Drug Saf. 2024;23(9):1069–77. 39262109 10.1080/14740338.2024.2386365

[40] Kobayashi T, Morimoto T, Ito K, Mawatari M, Shimazaki T. Denosumab vs. bisphosphonates in primary osteoporosis: a meta-analysis of comparative safety in randomized controlled trials. Osteoporos Int. 2024;35(8):1377–93. 38733394 10.1007/s00198-024-07118-0

[41] Wang B, Cheng X, Fu S, et al. Associations of serum 25(OH)D, PTH, and β-CTX levels with All-Cause mortality in Chinese Community-Dwelling centenarians. Nutrients. 2022;15(1):94. Published 2022 Dec 24. 36615752 10.3390/nu15010094 PMC9824656

[42] Scheidemann ER, Shajahan-Haq AN. Resistance to CDK4/6 inhibitors in Estrogen Receptor-Positive breast cancer. Int J Mol Sci. 2021;22(22):12292. Published 2021 Nov 14. 34830174 10.3390/ijms222212292 PMC8625090

